# The Possible Role of Placental Morphometry in the Detection of Fetal Growth Restriction

**DOI:** 10.3389/fphys.2018.01884

**Published:** 2019-01-08

**Authors:** Nastaran Salavati, Maddy Smies, Wessel Ganzevoort, Adrian K. Charles, Jan Jaap Erwich, Torsten Plösch, Sanne J. Gordijn

**Affiliations:** ^1^Department of Obstetrics and Gynecology, University Medical Center Groningen, University of Groningen, Groningen, Netherlands; ^2^Department of Obstetrics and Gynecology, Amsterdam University Medical Centers, University of Amsterdam, Amsterdam, Netherlands; ^3^Department of Anatomical Pathology, Sidra Medicine, Doha, Qatar

**Keywords:** FGR, IUGR, SGA, fetal growth restriction, intra uterine growth restriction, small for gestational age, placenta morphometry, birth weight

## Abstract

Fetal growth restriction (FGR) is often the result of placental insufficiency and is characterized by insufficient transplacental transport of nutrients and oxygen. The main underlying entities of placental insufficiency, the pathophysiologic mechanism, can broadly be divided into impairments in blood flow and exchange capacity over the syncytiovascular membranes of the fetal placenta villi. Fetal growth restriction is not synonymous with small for gestational age and techniques to distinguish between both are needed. Placental insufficiency has significant associations with adverse pregnancy outcomes (perinatal mortality and morbidity). Even in apparently healthy survivors, altered fetal programming may lead to long-term neurodevelopmental and metabolic effects. Although the concept of fetal growth restriction is well appreciated in contemporary obstetrics, the appropriate detection of FGR remains an issue in clinical practice. Several approaches have aimed to improve detection, e.g., uniform definition of FGR, use of Doppler ultrasound profiles and use of growth trajectories by ultrasound fetal biometry. However, the role of placental morphometry (placental dimensions/shape and weight) deserves further exploration. This review article covers the clinical relevance of placental morphometry during pregnancy and at birth to help recognize fetuses who are growth restricted. The assessment has wide intra- and interindividual variability with various consequences. Previous studies have shown that a small placental surface area and low placental weight are associated with a slower growth of the fetus. Parameters such as placental surface area, placental volume and placental weight in relation to birth weight can help to identify FGR. In the future, a model including sophisticated antenatal placental morphometry may prove to be a clinically useful method for screening or diagnosing growth restricted fetuses, in order to provide optimal monitoring.

## Background

**Figure 1:**
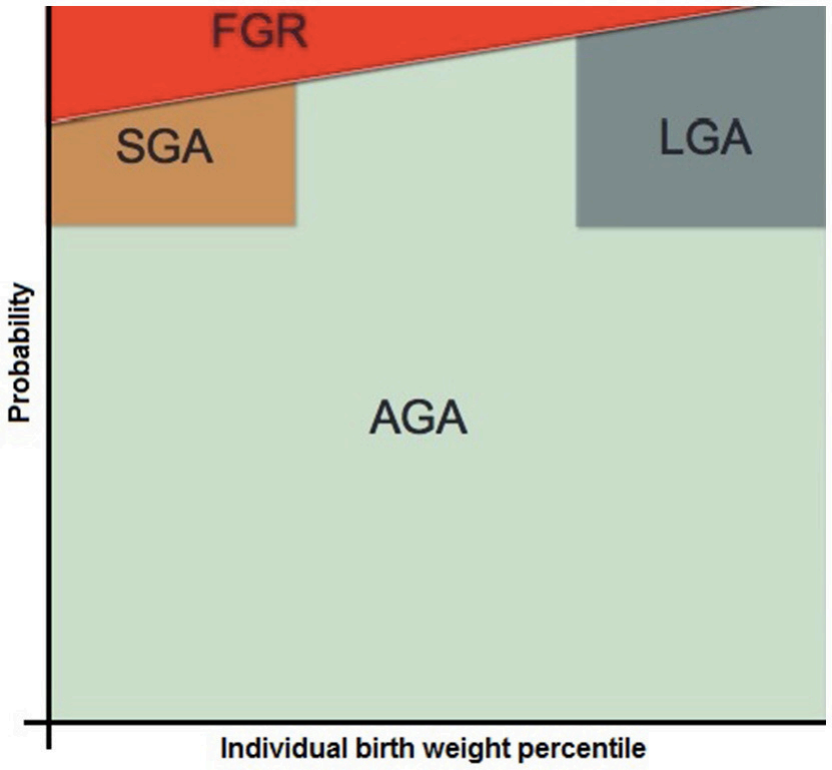
Schematic representation of the possible distribution of FGR within the total population consisting of SGA, AGA, and LGA fetuses at a certain gestational age. Another gestational age-period or population will most likely have a different distribution. FGR, fetal growth restriction; SGA, small for gestational age; AGA, appropriate for gestational age; LGA, large for gestational age [reproduced with permission from (Gordijn et al., 2018)].

The diagnosis of fetal growth restriction (FGR) has for long mainly be based on birth weight below a reference cut-off, most commonly the 10th percentile (p10) (Beune et al., 2018). Birth weight (BW) or estimated fetal weight (EFW) below p10 indicates that the BW or EFW is within the lowest 10% of BW compared to the reference population. This is in essence not FGR but small for gestational age (SGA). There are some important diagnostic issues with this misnomer. First, about 75% of fetuses who are SGA (and therefore many who are FGR) remain unrecognized until they are born and the diagnosis is made on the baby scale, postnatally (Monier et al., 2017; Beune et al., 2018), meaning some are severely compromised, exposed to potential long term sequelae, or even stillborn. Second, fetuses who are too small according to the intra uterine reference chart may be physiologically small and appropriate grown according to their individual growth potential (based upon their genetic and epigenetic inheritance at conception), and therefore not at risk from diseases related to FGR, but are exposed to unnecessary investigations for FGR. Third, many cases of growth restriction remain unacknowledged, when a baby or fetus is too small according to its *individual* growth potential, but not necessarily too small in the *population* based reference chart. Thus FGR overlaps with, but is not synonymous to, SGA (Zhang et al., 2010) (“SGA-FGR confusion”), as two overlapping distribution curves. It is self-evident that the incidence of growth restricted fetuses increases as EFW or BW percentiles decreases (Vasak et al., 2015). Yet, there is not a single cut-off above which all babies have grown appropriately, or below which none have grown appropriately for their individual biological growth potential. If SGA is used as the proxy for FGR in clinical practice, healthy SGA fetuses and neonates without FGR are prone to unnecessary monitoring intervention strategies and FGR fetuses and neonates who are FGR but not SGA remain unrecognized (“masked” FGR). Furthermore, if SGA is used as proxy for FGR in research, the study population is diluted by healthy small fetuses and newborns, hampering adequate association studies. It is estimated that in the SGA group, 60% were growth restricted, and 40% were constitutionally small (Figure 1) (Figueras and Gratacos, 2017). In this study, “constitutionally small” was defined as fetuses with moderately low BW (>3rd percentile) and normal placental function on both the fetal (normal cerebroplacental ratio) and maternal (normal uterine Doppler) sides. Severe growth restriction or evidence of placental dysfunction was defined as “growth restricted” (Figueras and Gratacos, 2017).

This study implies the relevance of appropriate, possibly easy to obtain, and cheap diagnostic tools to detect only those fetuses who are growth restricted because these are the fetuses who have an increased risk of adverse short- and long-term outcomes when not delivered in time (Jaddoe et al., 2014; Meher et al., 2015; Miller et al., 2016).

The causes of FGR can be divided into pre-utero-placental (e.g., maternal anemia, hypoxia, malnourishment), utero-placental (e.g., poor implantation, pre-eclampsia) and fetal conditions (e.g., fetal infection, some maldevelopments), and conditions like twin to twin transfusion. The utero-placental group appears the largest (ACOG Practice Bulletin no. 134: Fetal Growth Restriction, 2013), and the main focus to date has been on histopathological changes such as maternal malperfusion, villitis and more recently terminal villous hypoplasia. However, recently more focus has been made on examining the role of the gross examination of the placenta, with weight, shape, cord insertion. With better identification of the factors that are associated or causative for FGR, the baby who may or may not be small can still be identified as at risk for sequelae of FGR, based upon the severity of the changes. The issue is complex as the relationship is not straightforward, and several opposing forces are occurring. The placenta is not inert and does not grow purely as its genes dictate, but it appears to respond to the demands of the fetus and also the supply from the mother, appearing to adapt and compensate. It does this on a local level controlling blood flow through the stem villi with the arterial muscle, and globally, causing the increased placental resistance that in turn is identified by the Doppler studies. Furthermore, the fetus does the same with its redistribution of the blood, to the brain, at the cost of the liver glycogen and fatty tissue stores. There may also be a tradeoff on how much of the overall nutrient going to the conceptus is used for the placenta (to maximize uptake) or to the fetus, but usually the birth weight to placenta weight-ratio (BWP-ratio) increases in conditions with FGR. In addition, there are also well established differences between male and female fetuses.

Ideally for every fetus, all the relevant factors are assessed to give a rational approach to answer the question whether the fetus has reached its full growth percentile, based on the assessment of significant evidence of less than optimal maternal factors, uteroplacental factors including gross and histopathological placental examination, and fetal factors.

In this literature review we focus of the gross examination of the placenta and we aim to give an overview on the possible use of *placental morphometry* in recognizing fetuses and neonates with growth restriction, independent of their weight.

### Diagnosis of Fetal Growth Restriction

The most common pathophysiologic mechanism of FGR is placental insufficiency, with multiple underlying maternal, fetal and placental causes, resulting in insufficient nutrition and oxygen supply to the fetus (ACOG Practice Bulletin no. 134: Fetal Growth Restriction, 2013). As mentioned above, the diagnostic process is complicated by the “SGA-FGR confusion.” Placental insufficiency, placing fetuses at increased risk of hypoxia and malnourishment related morbidity, as well as stillbirth, is not restricted to those fetuses who are growth restricted and small, but also to those within normal weight ranges. The “masked” FGR fetuses in the subgroup of appropriate for gestational age (AGA) or even large for gestational age (LGA) fetuses, also experience placental insufficiency [assessed by umbilical artery pulsatility index (PI), middle cerebral artery PI, cerebroplacental ratio] (Morales Roselló et al., 2012). They show a slower growth trajectory during pregnancy, and are prone to the same risks. In addition, these fetuses have a further doubling of stillbirth risk compared to those fetuses with detected FGR, because no interventions to modify that risk are installed (Lindqvist and Molin, 2005; Gardosi et al., 2013). Clinically, these fetuses can only be recognized with sequential ultrasound measurements that show a decline in weight centiles, which is not routinely applied in general midwifery and obstetric practice. FGR, particularly early onset, is associated with histopathological changes through later onset may be histologically unremarkable (Mifsud and Sebire, 2014).

The major challenge of FGR is the diagnostic standard. In 2016 an international Delphi procedure among 56 experts on FGR was established to come to a consensus definition for both early (<32 weeks of gestation) and late FGR (≥32 weeks of gestation) (Gordijn et al., 2016). In this definition not only size parameters of the fetus but also parameters of placental function, either alone or in combination, are included and have been used widely since publication. The clinical applicability of these definitions in predicting adverse outcomes is yet to be assessed.

### The Role of Placental Size in Fetal Growth

Normal growth of the fetus is mainly dependent on normal placental function, with normal placental morphometry (size and shape) and normal structure. Impairments in placental development, including reduced placental size, or altered placental nutrient transport capability contribute to placental dysfunction (Zhang et al., 2015). Placental dysfunction attributable to structural fetal or genetic fetal defects share similar pathophysiologic pathways but are characterized by a different set of pathophysiologic features and are not included in this review. These factors contributing to placental dysfunction, as well as changes in the placental transport system, result in FGR.

To illustrate, it is known that transporter activity of system A amino acid uptake is reduced in placentas from FGR fetuses with abnormal umbilical artery Dopplers, and that it is also related to the severity of FGR (Glazier et al., 1997). The capability of the placenta to maintain sufficient nutrient supply is commonly described as “placental efficiency” and is described to be reflected by BWPW-ratio (Wilson and Ford, 2001). The increased risk of stillbirth may reflect less “placental reserve” with a high BWPW-ratio, where the fetus is running higher risk of stillbirth and yet maximizing its albeit constrained weight and growth. In animal studies, positive correlations were found between BWPW-ratio and placental uptake of nutrient transport system A amino acid uptake, indicating that nutrient transfer per gram placenta must have increased compared to low or normal BWPW-ratio (Hayward et al., 2016). However, in human studies these effects are less conclusive regarding the system A transporter. A low BWPW-ratio describes fetuses with a relatively large placenta (higher placental weight) compared to the birth weight, whereby the nutrient transfer is reduced per gram placenta.

### Antenatal Measurement of Placental Function

Prenatal screening for FGR in general obstetrical populations involves identifying risk factors for impaired fetal growth. When the fetus is identified to be at risk for FGR, sequential assessment of fetal size, either by anatomical reference points or by sequential ultrasound is executed. Actual fetal size reflects past placental function until that point in time, whereby “real time” placental function can be assessed *in vivo* by measurement of vascular resistance Doppler flows in the mother (uterine artery) or in the fetus (umbilical artery and middle cerebral artery) (Alfirevic et al., 2010). Doppler flow measurements enable the non-invasive detection of signs of placental insufficiency and fetal hemodynamic changes that occur during oxygen deprivation. During the course of normal, healthy, pregnancies, umbilical artery resistance decreases gradually throughout gestation, and increases with placental insufficiency (Unterscheider et al., 2013). Ghosh et al. suggested in their study on pregnancies complicated by suspected FGR fetuses that abnormal Doppler patterns of the uterine arteries could identify a fetus at increased risk, even in the presence of normal umbilical artery Doppler flow (Ghosh and Gudmundsson, 2009). Regarding fetal biometry, abdominal circumference is smaller in FGR fetuses due to depletion of abdominal adipose tissue as well as smaller liver size, because of reduced glycogen storage. As solitary parameter in the detection of FGR, measurement of the abdominal circumference is the most sensitive (Nardozza et al., 2017). In case of placental insufficiency and decreased oxygen and nutrients supply, the fetus redistributes the blood to the brain, at the costs of the liver glycogen and fatty issue stores, resulting in normal fetal brain growth and decline of growth of the abdominal circumference.

### Measurement of Serum Biomarkers

Biochemical biomarkers that reflect placental function and adverse pregnancy outcomes, including FGR, are increasingly subject of research. A systematic review conducted in 2013, assessed 53 studies investigating biomarkers that could potentially have a role in screening for FGR. They concluded that none of the 37 different biomarkers were sufficiently accurate to function as a predictor of FGR (Conde-Agudelo et al., 2013). However, different definitions of FGR were used in the evaluated studies in which the vast majority; 47 of the 53 studies, used SGA as a proxy for FGR. Gaccioli and colleagues, investigated whether an EFW below the 10th percentile in combinations with an elevated sFLIT1: PIGF ratio (at 36 weeks of gestation) was predictive for adverse pregnancy outcomes. They showed that this combination was strongly predictive for delivering a SGA infant (birth weight < 10th centile) plus perinatal morbidity and/or preeclampsia (Gaccioli et al., 2018a). In a subsequent study, elevated FLIT1:PIGF ratio was combined with low abdominal circumference growth velocity (ACGV), and a composite measure generated by, the earlier mentioned, Delphi procedure (Gordijn et al., 2016), described as indicators of FGR (Gaccioli et al., 2018b). They found that at a gestational age of 28 as well as 36 weeks, the positive predictive value of ultrasonic screening for the delivery of a SGA infant with complications was doubled when it was combined with biochemical markers compared to the ultrasonic screening method alone. The relation with placental morphometry has not been examined in these studies.

## Clinical Relevance of Antenatal and Postnatal Assessment of placental morphometry

In current clinical practice, placental morphometry is only routinely investigated and described at pathological examination, after delivery. In general, placentas are only routinely investigated in case of some adverse pregnancy outcomes. However, examination of placental morphometry, both in the antenatal and postnatal phase, can possibly disclose information for the detection of fetal growth restriction.

### Antenatal Assessment of Placental Morphometry

Evaluation of the placenta during pregnancy is usually only performed to assess the location of the placenta or to diagnose placental adhesion disorders (e.g., *placenta praevia, placenta increta, placenta bilobata*). Antenatal assessment of placental morphometry, alone or in relation to fetal size, is not routinely performed but may improve the identification of fetuses at risk for adverse outcomes caused by placental insufficiency. The theoretical advantage of antenatal assessment compared to postnatal assessment of placenta morphometry is obvious; relevant information from antenatal assessment does not only apply to the newborn or future pregnancies, but could also be of vital importance in the current pregnancy. It can allow the clinician to tailor monitoring- and intervention strategies to reduce the risk of adverse outcomes.

Unfortunately, literature regarding the clinical relevance of antenatal assessment of placental morphometry is still scarce. In this paragraph, we will summarize the existing literature on placental morphometry assessment during pregnancy with both ultrasound and MRI.

#### Antenatal Assessment of Placental Morphometry With Ultrasound

Measurements of placental diameter and thickness, using two-dimensional ultrasound, have been used as indicator of high-risk pregnancies and correlates with birth weight (Afrakhteh et al., 2013). Several studies have investigated these ultrasound measures in relation to SGA (birth weight < p10), and showed that placental diameter and thickness are lower in SGA fetuses (Habib, 2002; Afrakhteh et al., 2013; Mathai et al., 2013; Schwartz et al., 2014). In addition, Schwartz and colleagues had the aim to combine early, direct ultrasound assessment of the placenta with other markers of placental development, such as mean of the uterine artery Doppler PI, to identify pregnancies delivering SGA infants (Schwartz et al., 2014). Placental volume, placental quotient (PQ = placental volume/gestational age), and mean placental diameter were significantly smaller in fetuses in the SGA group, compared to the AGA group. This indicates that a smaller placental mass is associated with SGA (Heinonen et al., 2001; Chisholm and Folkins, 2016).

On the other hand, the placental morphology index [defined by mean placental diameter divided by placental quotient (PQ)] was significantly higher in the SGA group, demonstrating a closer association between slower fetal growth and a relatively wide and flat placenta, rather than a relatively thick placenta (Schwartz et al., 2014).

Studies that used FGR as outcome variable (Table 1) showed that abnormal placental shape (placental thickness > 4 cm or >50% of placental length) were predictive for experiencing FGR (Viero et al., 2004; Toal et al., 2007, 2008). In addition, Proctor et al. showed that FGR was associated with small placental size (linear placental length < 10 cm), in a group of women with low first trimester PAPP-A ( ≤ 0.30 multiples of median) (Proctor et al., 2009).

**Table 1:** Literature overview of antenatal placental morphometry assessment with ultrasound in relation to FGR (markers).

**Author, year, country**	**Study type and population**	**FGR definition**	**Determinant(s)**	**Outcome**	**Results**
Viero et al., 2004, Canada	Prospective 60 SP with ARED flow velocities in UmA.	EFW < p10 and - Elevated HC/AC - Amniotic fluid index (AFI) < 10 cm	PL, PT, cord insertion Small/thick placenta: max PT > 4 cm, in absence of uterine contraction OR >50% of PL	Pregnancies with ARED in UmA.	Ultrasound was accurate in identifying lateral or marginal cords (sensitivity 86%, PPV 71%; *p* < 0.0001) Ultrasound examination of the placenta and its maternal blood supply may contribute to the perinatal management of pregnancies with high risk of perinatal morbidity/mortality.
Toal et al., 2007, Canada	Prospective 212 high risk* SP	EFW < p10 delivered at < 34 wks and - Ultrasound examinations demonstrating reduced fetal growth - AFI < 10 cm - ARED in UmA	Abnormal placental shape: PT > 4 cm or >50% of PL. Abnormal placental cord insertion. Placental texture. GA: 18–23 wks	FGR	The odds of the development of FGR were sign. less in women with all normal test results. Combining those women with two (*n* = 21) of three (*n* = 3) abnormal test results together predicted 14 of 19 severe FGR.
Toal et al., 2008, Canada	Prospective 60 high risk* SP with abnormal UtA Doppler flow at GA 19–23 weeks	EFW < p10 delivered at < 34 wksand-Ultrasound examinations demonstrating reduced fetal growth- AFI < 10 cm- Abnormal UmA Doppler waveforms	Abnormal placental shape (PT/PL ratio of >0.5 or PT of >4 cm)	FGR	Women with abnormal placental shape had higher odds of FGR (odds, 4.7; 95% CI, [1.6–14.1]). Combined abnormal UtA Doppler flow and placental dysmorphologic condition before fetal viability identifies a subset of women who are at risk for adverse outcomes.
Proctor et al., 2009, Canada	Prospective 90 SP with first trimester PAPP-A ≤ 0.30 multiples of median.	EFW < p10 with UmA PI >p95 or ARED in UmA, and - Serial fetal biometry demonstrating growth failure.- AFI < 5 cm	Placental size (linear placental length). GA: 18–24 wks	FGR, preterm delivery before 32 weeks, stillbirth	FGR was sign. associated with small placental size (linear placental length < 10 cm), in group of women with low PAPP-A.

* defined as “significant medical and/or obstetric risk factors for hypertensive disease/placental insufficiency.”

*AC, abdominal circumference; AFI, amniotic fluid index; ARED, absent or reversed end diastolic; EFW, estimated fetal weight; FGR, fetal growth restriction; GA, gestational age; HC, head circumference; PAPP-A, pregnancy-associated plasma protein A; PL, placental length; PPV, positive predictive value; PT, placental thickness; SP, singleton pregnancies; UmA, umbilical artery; UtA, uterine artery; wks, weeks*.

In order to assess placental morphometry during pregnancy with ultrasound, sonographic reliability of placental measurements has to be adequate. In this regard, a couple of limitations have to be addressed. First, there are no existing, *in vivo*, ultrasound reference charts of normal placental size. Although Higgins et al. described that the estimated placental biometry and volume during pregnancy are correlated with their measurements at postnatal assessment, they are not equal (Higgins et al., 2016). *In vivo* measurements, performed within 7 days before delivery, of placental length and width, and 3D placental volume measurements were smaller compared to *ex vivo* measurements (Higgins et al., 2016). Placental depth and 2D placental volume measurements were found to be larger compared to their *ex vivo* correlates. These differences are probably caused by the collapse of intervillous space due to loss of maternal blood flow after birth and less stretching of the placenta due to the loss of intrauterine pressure from amniotic fluid and the baby volumes after birth. Azpurua et al. described that placental weight could be accurately predicted by 2D ultrasound with volumetric calculation (Azpurua et al., 2010). Second, intra- and inter-observer variability play a much bigger role with *in-vivo* sonographic measurements than *ex-vivo*, real life measurements (Higgins et al., 2016). Higgins et al. investigated the intra- and inter-observer variability between observations for placental measurements length, width, depth and volume performed by 2D ultrasound. The variability in measurements (intra- and inter-) was suboptimal with no intraclass correlation coefficient >0.75 (Higgins et al., 2016). More recently, a new technique was established for estimating placental volume from 3D ultrasound scans through an semi-automated technique (Looney et al., 2018). In this study, placental volume of 2,393 pregnancies was assessed by three operators on the one hand, and this semi-automated tool on the other hand. The clinical utility of placental volume was tested by looking at prediction of SGA at term. Results showed good similarity between the operators and the tool, and almost identical clinical results for the prediction of SGA (Looney et al., 2018).

#### Antenatal Assessment of Placental Morphometry With Magnetic Resonance Imaging

Magnetic resonance imaging (MRI) is an established, safe method of imaging during second and third trimester of pregnancy, but currently mainly used for fetal imaging (Wang et al., 2012a,b; Bulas and Egloff, 2013). The advantages of MRI compared to ultrasound, are the more accurate measurements of anatomical volume and the higher soft tissue contrast, and thus it has specific strengths in detecting abnormal placental morphometry. Furthermore, it has a larger field of view and, other than ultrasound, it is not dependent on its ability to penetrate tissue.

Reference values of placental volume by MRI measurements throughout gestation of healthy pregnancies, although in relatively small sample size, have been studied and are available now (Duncan et al., 2001; Langhoff et al., 2017; León et al., 2018). Current research on placental imaging is much more focused on the more advanced techniques of functional MRI (fMRI), rather than assessment of placental morphometry with conventional MRI. These fMRI techniques, and their implications for diagnosing FGR, are not in the scope of this review but are described in the reviews of Avni et al. (2015) and Siauve et al. (2015).

Although current research focuses more on the possibilities of fMRI, five studies specifically investigated the placenta morphometry measurements with MRI in relation to FGR, or markers of FGR (Table 2). The study of Derwig et al. was the only one that used SGA as outcome rather than FGR, and showed that small placental volume is predictive for delivering a SGA-neonates, which is in line with the findings from other (ultrasound) studies and could be a physiological phenomenon (Derwig et al., 2011). They also described that small placental volume was significantly associated with higher PI of the uterine artery, a marker of FGR. Increased uterine artery PI is thought to reflect defective trophoblast invasion, which could result in reduced placental growth (Arakaki et al., 2015).

**Table 2:** Literature overview of antenatal placental morphometry assessment with MRI in relation to FGR (markers).

**Author, year, country**	**Study type and population**	**FGR definition**	**Determinant(s)**	**Outcome**	**Results**
Damodaram et al., 2010, United Kingdom	Prospective 48 SP **A:** 20 FGR fetuses (1) *n* = 3, (2) *n* = 3, (3) *n* = 10, (4) *n* = 1, (5) *n* = 3 **B**: 28 controls	EFW < p5 and (1) PI UmA >p95 (2) PI UmA>p95 and PI MCA < p5 (3) AEDF in UmA (4) REDF in UmA (5) Absent or reversed “a” wave in DV and/or pulsatility in UV	PV, max PT, PT/PV ratio GA: 20–38 wks	Morphometry determinants in relation to: - Group A vs. B - Severity of FGR - Fetal and neonatal mortality	Sign. increase in max PT/PV ratio in group A Sign. correlation: max PT/PV ratio –severity FGR, PV–EFW, PV–severity of FGR Association: increase in max PT/PV ratio >p95–fetal and early neonatal mortality PV remained sign. smaller in group A
Dahdouh et al., 2018, United States of America	Prospective 49 SP **A:** 43 FGR fetuses **B:** 46 controls	EFW < p10 and -Abnormal Doppler of fetal vessels - Asymmetric growth with lagging AC	PV, PT, PL icw textural features used in machine learning frameworks GA: 18–39 wks	Identification of the FGR pregnancies	The proposed machine-learning based method using shape features identified FGR pregnancies with 86% accuracy, 77% precision and 86% recall.
Ohgiya et al., 2016, Japan	Prospective 50 SP **A:** 25 FGR fetuses **B:** 25 controls	No definition given	PT, PSA, PV GA: 19–38 wks	Morphometry determinants in group A vs. B	Sign. lower mean PSA and PV in group A compared to group B. Sign. higher mean PT in group A compared to group B.
Andescavage et al., 2017, United States of America	Prospective 114 SP **A:** 35 FGR fetuses **B:** 79 controls	EFW < p10 and - UmA PI >p95 or CPR < 1 - AC lagged HC > 1 week	PV GA: 18–40 wks	Morphometry determinant in relation to: - group A vs. B - UmA Doppler, UtA Doppler, MCA Doppler, CPR	Sign. lower mean PV in group A vs. B Sign. lower mean PV in subgroup of group A with abnormal UmA Doppler. no association between: PV–UtA Doppler, PV–MCA Doppler, PV–CPR
Derwig et al., 2011, United Kingdom	Prospective 83 SP	Not applicable	PV GA: 24 - 29 wks	Morphometry determinant in relation to UtA Doppler and BW-centile	Median PV was sign decreased in pregnancies delivering a SGA-neonate (< p10) PV is sign related to the degree of uterine perfusion reflected in the PI of the UtA.

*AC, abdominal circumference; CPR, cerebroplacental ratio; DV, ductus venosus; EFW, estimated fetal weight; FGR, fetal growth restriction; GA, gestational age; HC, head circumference; icw, in combination with; MCA, middle cerebral artery; PI, pulsatility index; PL, placental length; PSA, macroscopic placental surface area; PT, placental thickness; PV, placental volume; sign, significantly; SP, singleton pregnancies; UtA, uterine artery; UmA, umbilical artery; UV, umbilical vein; wks,weeks*.

Three studies (Damodaram et al., 2010; Ohgiya et al., 2016; Andescavage et al., 2017) investigated placental morphometry measurements in a FGR population compared to healthy controls. Although different definitions of FGR have been used (see Table 2), they all showed significantly reduced placental volume in the FGR population compared to the healthy pregnant population. Furthermore, Damodaram et al. showed that the placental volume remained significantly smaller throughout gestation in the FGR group, and that a lower placental volume was also associated with the severity of the FGR (detailed information on the severity subgroups can be found in Table 2) (Damodaram et al., 2010). Finally, Andescavage et al. described that the placental volume was significantly lower in a subgroup of the FGR-population with abnormal umbilical artery Doppler (Andescavage et al., 2017). Higher mean placental thickness, lower macroscopic placental surface area and increase in max placental thickness/placental volume (PT/PV) ratio, were placental morphometry parameters that were significantly associated with FGR (Damodaram et al., 2010; Ohgiya et al., 2016).

Further substantiation of the relevance of the max PT/PV ratio was shown by the significant correlation found between a higher max PT/PV ratio and the severity of the FGR, and the association with fetal and early neonatal morbidity in case of an increase of the max PT/PV ratio above the 95th percentile for gestation (Damodaram et al., 2010).

The last and most recent study of Dahdouh et al. had a slightly different study design. In this study placenta morphometry, although in combination with placental textural features computed on 3D MRI images, were used in two machine learning frameworks to predict FGR and BW for both healthy and FGR fetuses (Dahdouh et al., 2018). This semi-automated framework was able to detect FGR-fetuses with a diagnostic accuracy of 86%, sensitivity of 86% and a specificity of 87%. In line with the other four studies, placental volume was one of the most important features for identification of FGR. Although this study had a small sample size (*n* = 80), these results are promising, outperforming the current standard clinical tools for diagnosing FGR. Although MRI is increasingly used during pregnancy, especially at advanced gestation or in obese women (Millischer et al., 2013), availability is limited and costs are high. Therefore, current clinical use of MRI in the assessment of placental morphometry is very limited.

### Postnatal Assessment of Placental Morphometry

After birth, standard placental measures are placental disk shape, diameter, surface area, disk thickness, weight, location of umbilical cord insertion site relative to the edge of the placental disk, and placental weight in relation to birth weight (Khong et al., 2016). It is advised to use placental weight trimmed of extraplacental membranes and umbilical cord (Khong et al., 2016). Inconsistencies in preparation of the placenta before weighing remains in different studies. Therefore, it is important to check whether trimmed or untrimmed placental weights are used, as for direct comparisons between absolute placental weights, values should be standardized to trimmed placental weight (Leary et al., 2003).

There is increasing evidence that features of placental gross morphology are linked biologically to the functional capacity of the placenta (Burton et al., 2016), but it has received little clinical interest. The reason for this is the timing of investigation of the placenta: pregnancy conditions have either developed or not, and intra-uterine problems already have taken place before the possibility to investigate the placenta. However, postnatal morphology studies of the placenta give the opportunity to help in finding the neonate who suffered undetected growth restriction and should be monitored more closely during postnatal care. It is thus important to focus on the possible clinical relevance of placental morphometry in retrospectively diagnosing impaired growth.

#### Postnatal Placental Morphometry in Relation to Ultrasound Markers of Fetal Growth Restriction

It has been shown that utero-placental blood flow and fetal growth can be related to the gross morphometry of the placenta (Salavati et al., 2016). Small placental area and low placental weight were associated with, respectively, higher uterine and higher umbilical artery PI. Both placental area and weight were associated with a slower fetal ACGV (Salavati et al., 2016). The circularity of the placenta was associated with the uterine artery, but not the umbilical artery, flow velocity waveform. These results show that size and shape of the placenta are depending on the vascular function of the placenta from both the maternal and fetal side. Although some studies have focused on the relationship between ultrasound measurements (e.g., fetal biometry, Doppler flow velocity waveforms) and adverse pregnancy outcome (Ghosh and Gudmundsson, 2009; Sovio et al., 2015), more literature on the relationship with postnatal placental morphometry is lacking.

##### Birth weight to placenta weight-ratio in relation to ultrasound markers

Research showed that not only the size and weight of the placenta, but also the weight of the placenta in relation to birth weight was associated with both umbilical and uterine artery PI (Salavati et al., 2018). Specifically, high BWPW-ratio was associated with both higher umbilical artery PI (26 weeks of gestation) and higher uterine artery PI (20 weeks of gestation), two markers of decreased placental function. Low BWPW-ratio was not associated with either umbilical or uterine artery PI, however it was with maternal and neonatal morbidity (Salavati et al., 2018). Decreased placental function may sound contradictory, since placentas in the group of high BWPW-ratio can also be seen as very efficient. It might be plausible that the relatively small placentas in the group of high BWPW-ratio work at their maximum function capability for that volume, and that the birth weight actually could have been higher with higher placental volume. With this said, we would expect high BWPW-ratio to be related to adverse postnatal outcome related to starvation caused by too relatively small placentas, which was not shown by recent research (Salavati et al., 2018). This might be the result of intervention bias: those cases with high umbilical and uterine artery PI might have experienced an earlier induction of labor, resulting in lower neonatal morbidity (Gibson et al., 2014). Another explanation might be the role of placental surface area. Those placentas in the group of low BWPW-ratio might be thicker but have a small macroscopic placental surface area, resulting in a less efficiently exchange process of oxygen, nutrients, and fetal waste products. In the group of high BWPW-ratio the reverse might be true; within this group the placentas might have a large macroscopic placental surface area, but are really thin, explaining the lower placental weight.

#### Postnatal Placental Morphometry in Relation to Birth Weight and Fetal Growth Restriction

Various studies have looked at the relationship between postnatal placental morphometry and birth weight. These studies showed that low birth weight was associated with lower placental weight and volume, and a smaller placental area (Balihallimath et al., 2013; Kowsalya et al., 2013). Balihallimath et al. studied the relationship of placenta morphometry more specifically in different birth weight groups, classified by gender (Balihallimath et al., 2013). In the groups with birth weight less than 3,000 g, the surface area of the placenta was smaller in male babies compared to female babies. When birth weight exceeded 3,000 g, the surface area was larger in male babies (Balihallimath et al., 2013). In addition, it has been described that male babies have a higher birth weight compared to female babies, but have the same placental weight (increased BWPW-ratio) (Eriksson et al., 2010; Macdonald et al., 2014). Male babies also have a higher perinatal mortality (Drevenstedt et al., 2008), rather than more efficient they may also be just the fetus that runs the risk of increased mortality to maximize growth. We think the placenta has a functional reserve to cope with extra demands, which explains why many stillbirths have pre-existing injury (Gardosi et al., 2013).

Although a small placenta suggests reduced reserve, the association between low birth weight and low placental weight and size, could potentially be physiological (small baby, small placenta) therefore statements regarding the association between placental morphometry and pathological, low or high, birth weight, require an investigation of proportionality, of the relationship of placental morphometry with BWPW-ratio. Also the site of the umbilical cord insertion has been linked to birth weight (Yampolsky et al., 2009; Haeussner et al., 2013; Kowsalya et al., 2013). Kowsalya et al. indicated that the cord insertion was more often eccentric or marginal in the group of infants with low birth weight (Kowsalya et al., 2013). Yampolsky et al. pointed out that this central insertion influences placental efficiency positively (Yampolsky et al., 2009). Conflicting results were published by Haeussner et al. who reported that the location of the cord insertion in relation to the edge of the placental disk did not correlate with birth weight, and eccentric cord insertion did not necessarily compromise efficiency of the normal human placenta (Haeussner et al., 2013). Furthermore, they reported that parameters regarding the form of the placenta (e.g., diameter, thickness, roundness, eccentricity of the cord insertion) correlate with both birth weight and placental weight (Haeussner et al., 2013).

It has been proposed that FGR and morphologic changes of the placenta, are caused by impaired placental perfusion, due to reduced placental vascular bed in chronic fetal hypoxia, which causes oxidative stress of the fetal vasculature (Kingdom and Kaufmann, 1997; Kuzmina et al., 2005). Junaid et al. investigated micro and microvasculature of placentas from normal and FGR pregnancies, and observed hyposvascularity in the peripheral lobules of placentas from FGR pregnancies (Junaid et al., 2014). Another aspect that may result in altered morphometry, is the fact that FGR related hypoxia influences angiogenesis via various growth factor receptors (Mayhew et al., 2004). Three studies were found that investigated the relationship between postnatal placental morphometry and FGR (Egbor et al., 2006; Mayhew et al., 2007; Almasry and Elfayomy, 2012) (Table 3).

**Table 3:** Literature overview of postnatal placental morphometry assessment in relation to FGR (markers).

**Author, year, country**	**Study type and population**	**FGR definition**	**Determinant(s)**	**Outcome**	**Results**
Egbor et al., 2006United Kingdom	Prospective **A:** 17 FGR **B:** 16 PE-FGR **C:** 16 controls	- Clinical evidence of suboptimal growth - Ultra sonographic evidence of deviation from appropriate growth percentile - BW < p10	PV, PW	FGR	FGR was associated with a significant reduction in PV and PW
Mayhew et al., 2007, United Kingdom	Prospective **A:** 5 FGR **B:** 5 PE-FGR **C:** 9 controls	- Deficient fetal growth on ultrasound scans - IBR < p10	PSA	Morphometry determinant in the different groups	FGR (with or without PE) was associated with a reduced PSA
Almasry and Elfayomy, 2012, Saudi Arabia	Case-control **A:** 50 FGR **B:** 25 controls	EFW < p10 icw two criteria: - Abnormal UmA Doppler - Oligohydramnios; AFI < 5 - Asymmetric growth; HC/AC ratio	PD, PW, “placenta co-efficient” (PW/BW).	FGR	Significant reduction in PD and PW in FGR group as compared with controls. Placental coefficient greater in FGR group.

*AC, abdominal circumference; BW, birth weight; EFW, estimated fetal weight; FGR, fetal growth restriction; HC, head circumference; IBR, individualized birth weight ratio (is calculated using factors including fetal gender, GA, parity, ethnic origin and maternal age, height and booking weight); icw, in combination with; PE, preeclampsia; PD, placental diameter; PSA, macroscopic placental surface area; PV, placental volume; PW, placental weight; UmA, umbilical artery*.

These three studies classified FGR based on deficient fetal growth on ultrasound scans and EFW less than 10th centile. They found that FGR was associated with changes in placental morphometry such as decreased surface areas, decreased placental diameter, and decreased placental volume and placental weight (Egbor et al., 2006; Mayhew et al., 2007; Almasry and Elfayomy, 2012).

## Future perspectives

As described, various studies have focused on the relationship between placental morphometry and fetal growth and birth weight. Unfortunately, only limited research has been performed focusing on associations between placental morphometry and FGR. The often seen “SGA-FGR confusion” in FGR studies will lead to weaker associations with any effective screening test, including placental morphometry imaging. Although cheap and easy to obtain, the postnatal placenta will only aid the diagnosis of FGR in retrospect. This could result in altered management by less monitoring in healthy SGA and more monitoring in FGR, regardless of birth weight. Although we expect that aspects of placental morphometry can play a role in the diagnostic process of FGR, mainly postnatally, additional components, as part of a multiparameter model, might need to be taken into account with the aspects of placental morphometry. A small placenta, or abnormally flat or thick placenta may prompt review for assessment of the baby and the histology of the placenta to see if other findings to suggest maternal malperfusion or other pathology is present.

Further use of placenta morphometry in the diagnostic process of FGR can be explored by for example BWPW-ratio in relation to abdominal circumference growth velocity (ACGV), macroscopic placental surface area (and placental volume) and placenta serum biomarkers. As suggested in a recent paper about screening for fetal growth restriction (Gaccioli et al., 2018a), it is expected that future screening tests for FGR will include several measurements, which are obtained from both imaging procedures and measurements of biomarkers. For the development of such a model it is essential that every single parameter is measured and scaled in the association with FGR, in order to generate consistent associations. Regarding imaging procedures, research has shown that placental imaging through MRI might be of clinical use in predicting FGR. Nowadays, MRI is already in use for fetal imaging, so possibly placental imaging can be used in the near future.

## Conclusions

It is of great importance that clinically useful, and easy to perform, methods will be generated in order to improve the antenatal and postnatal screening for, and diagnosis of, fetuses with FGR who have increased risk on adverse pregnancy outcomes. In this literature review we intended to give an overview on the clinical relevance of placenta morphometry in the detection of FGR. In current clinical practice, antenatal placental imaging is difficult, and the placenta is not routinely examined after birth, nor in a standardized way, despite the possible value in several parameters of impaired growth of the fetus. Future research can focus on the relationship between placental morphometry, FGR and its complications, to improve screening for FGR, and to determine the biological pathways that can be linked to placental dysfunction, in a group of optimally phenotyped cases of FGR. With this, placental morphometry might be implemented in clinical practice, possibly as part of a multiparameter model.

## Author Contributions

All authors made substantial contributions to the conception of this review and the critical appraisal of the literature summarized herein. NS and MS generated the initial draft of the manuscript, and this was critically revised by SG, WG, AC, TP, and JE. All authors approved the final version and submission of the article.

### Conflict of Interest Statement

The authors declare that the research was conducted in the absence of any commercial or financial relationships that could be construed as a potential conflict of interest.

## Abbreviations

AGA: appropriate for gestational age
BW: birth weight
CPR: cerebroplacental ratio
EFW: estimated fetal weight
FGR: fetal growth restriction
GA: gestational age
GV: growth velocity
HC: head circumference
LGA: large for gestational age
MRI: Magnetic Resonance Imaging
PAPP-A: pregnancy-associated plasma protein A
PI: pulsatility index
PIGF: placenta growth factor
PT: placental thickness
PV: placental volume
PW: placental weight
PQ: placenta quotient (= PV/gestational age)
sFLIT1: soluble fms-like tyrosine kinase 1
SGA: small for gestational age
UmA: umbilical artery
UtA: uterine artery.
